# Modified Fixed Loop-in-Loop Technique in Mitral Valve Repair for Children

**DOI:** 10.1016/j.atssr.2023.12.003

**Published:** 2023-12-23

**Authors:** Kunihiko Joo, Yoshie Ochiai, Yuma Motomatsu, Jun Muneuchi, Shigehiko Tokunaga

**Affiliations:** 1 Department of Cardiovascular Surgery, JCHO Kyushu Hospital, Kitakyushu City, Japan; 2 Department of Pediatric Cardiology, JCHO Kyushu Hospital, Kitakyushu City, Japan

## Abstract

For adults, the standard procedure for mitral valve repair of Carpentier classification type II mitral regurgitation is reconstruction with artificial chordae. In children, placement of artificial chordae of precise length between the papillary muscle and prolapsed mitral leaflet in the restricted mitral subvalvular space is technically difficult. We successfully performed mitral valve repair in 3 pediatric patients using a modified fixed loop-in-loop technique.

In pediatric patients, determination of the precise length of the chordae in the restricted mitral subvalvular space is technically difficult. Herein, we performed mitral valve repair in pediatric patients using a modified fixed loop-in-loop (FLIL) technique consisting of primary mini-loops and a second loop. This method allows accurate chorda length placement while minimizing attachment to the papillary muscle.

## Technique

Detailed transesophageal echocardiography was performed to measure the approximate chorda length based on the normal height of the prolapsed valve leaflet and papillary muscle. A video of the operation performed in this study is available (Video). To create the primary mini-loop, two 19-gauge needles were used as the core, and a single Gore-Tex CV-5 suture (W. L. Gore & Associates) was threaded around the circumference and ligated three times, repeating the process according to the number of anchor mini-loops that were to be created (Figure A). The size of the expanded polytetrafluoroethylene (ePTFE) suture should be selected on the basis of the patient's weight, with size reduction to a Gore-Tex CV-6 suture considered for weights ≤10 kg. Mini-loop–fixing ligation was performed by passing the contralateral needles through the knot spaces; thereafter, one 19-gauge needle was removed (Figure B). The primary mini-loop was sutured to the left ventricular papillary muscle where the artificial chorda had a pair of ePTFE pledgets. In cases in which artificial chordae were to be reconstructed from the bilateral papillary muscles, 2 primary-loop pairs were formed and sutured to the anterior and posterior papillary muscles. A Gore-Tex CV-5 suture was threaded through the primary mini-loop, and the second loop was formed by a double-armed mattress suture passing through the free edge of the prolapsed leaflet from the left atrial to left ventricular side to create a deep coaptation zone. The second loop was tightened with a vascular dilator or rubber tube as a spacer with a diameter matching the following formula: approximate chorda length × (2/pi) (Figures C, D). Ligation of the second loop with appropriate strength is important to avoid damaging the valve leaflets. A regurgitation test was performed with cardioplegic solution, and if readjustment of the length of the artificial chordae was necessary, the second loop at the lesion site was resected. If an artificial chorda required shortening, a smaller size was used, whereas if lengthening was required, a larger size was used.

**Figure:**
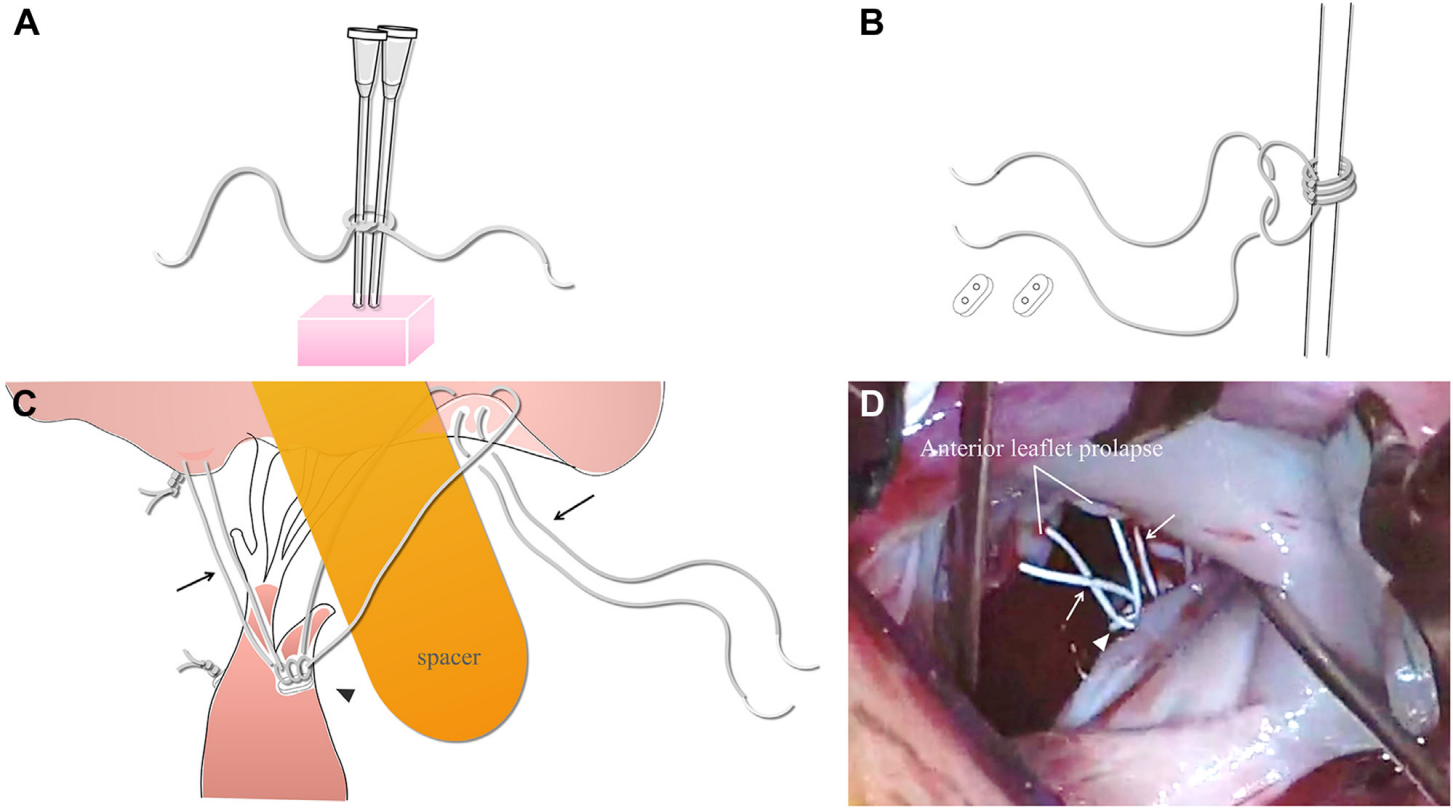
Chordal reconstruction by the modified fixed loop-in-loop method. (A) A single expanded polytetrafluoroethylene (ePTFE) suture ligated around two 19-gauge needles to determine the number of loops required. (B) Primary loop-fixing ligation. (C) The primary loop sutured to the papillary muscle with a pair of ePTFE pledgets. A double-armed ePTFE suture passes through the prolapsed leaflet, and a knot is fixed with an adjusted vessel dilator or rubber tube as a spacer (black arrow, secondary loop; black arrowhead, primary mini-loop sutured to papillary muscle). (D) Multiple neochordae created with modified loop-in-loop technique (white arrow, secondary loop; white arrowhead, primary mini-loop sutured to papillary muscle).

## Comment

Artificial chorda reconstruction with ePTFE sutures is widely used in mitral valve repair for mitral regurgitation caused by mitral valve prolapse in adults,^1^ and many excellent results are reported with the standard loop technique.^2^ Although this technique for artificial chorda reconstruction is performed in pediatric patients as well,^3^ precise selection of the suture site and adjustment of length in the narrow mitral subvalvular space become especially crucial. We performed mitral valve repair in adult patients using the FLIL technique of artificial chorda reconstruction and obtained good results.^4^ The FLIL technique has the advantage of minimizing suture-related damage to the papillary muscle by fixing the primary loop to the papillary muscle in a single pass and accurate ligation of slippery ePTFE second-loop sutures with a spacer; this process compensated for the difficulty of the original loop technique. In this technique, we used a 1-mm-diameter mini-loop as the anchoring primary loop to allow fine adjustment of the chorda length by the second loop in smaller pediatric patients. In our method, the length of the second loop for the artificial chordae can theoretically be adjusted with an accuracy of <1-mm increments by selecting a spacer in 0.5-mm increments: 0.5 mm × circumference ratio/2.

Another important concomitant procedure for adult mitral valve repair is annuloplasty with use of a mitral ring appropriate for the lesion. However, in most pediatric cases, a custom-made ring is unavailable, resulting in unbalanced annuloplasty. In addition, a patient with coexisting left-to-right shunt disease would experience changes in the dimensions of the left ventricle after concomitant left-to-right shunt lesion repair. In these cases, the preoperative chorda length assessed by transesophageal echocardiography may not be applicable, forcing length readjustment of the artificial chorda. The advantage of the FLIL technique is that length of the artificial chorda can be readjusted by simply resecting the second loop and readjusting the length of the second loop without additional damage to the papillary muscle.

Another challenge specific to pediatric patients is the need for longer durability along with their somatic growth. Satisfactory long-term results observed during 15 years of mitral valve repair with ePTFE chordae have been reported.^3^^,^^5^ However, lifetime durability remains unknown. A less common complication requiring mitral regurgitation due to ruptured chordae or ePTFE chordae calcification has previously been reported.^6^ During mitral valve re-repair, readjustment of the artificial chordae is crucial,^7^ in which the FLIL technique allows reconstruction of the neochordae with only a second-loop adjustment.

In our study, we treated 3 pediatric patients aged 1 to 7 years, weighing 7 to 17 kg, using the modified FLIL technique. Kay-Reed annuloplasty was performed in 2 cases, and autologous pericardial patch augmentation of the anterior leaflet was performed in 1 case, with additional indentation closure as required. Two to 4 artificial chordae were reconstructed with Gore-Tex CV-5 sutures for the patients. Two patients underwent multiple cardiopulmonary bypasses to readjust the second-loop length and site of construction, 1 of whom had a large concomitant atrial septal defect wherein the postoperative increase in left ventricular diameter caused the difference from the preoperative assessed length. Such re-repair of the mitral valve was feasible by readjustment of the second-loop length. During the observation periods spanning 0.8 to 3.4 years, the mean pressure gradient across the mitral valve remained <4 mm Hg, and no more than a mild mitral regurgitation was observed in all patients.

The modified FLIL technique can be applicable in all pediatric patients who are eligible for mitral valve repair by the loop technique. This method allows repeatable and accurate adjustment of the length of the artificial chorda. Further observation regarding the long-term durability after growth of the patient is needed in future research.
